# Polymeric nanoparticles containing diazepam: preparation, optimization, characterization, in-vitro drug release and release kinetic study

**DOI:** 10.1186/s40580-016-0061-2

**Published:** 2016-03-01

**Authors:** Sarvesh Bohrey, Vibha Chourasiya, Archna Pandey

**Affiliations:** Department of Chemistry, Dr. Harisingh Gour University, Sagar, Madhya Pradesh 470003 India

**Keywords:** Biodegradable polymer, Diazepam, Emulsion solvent evaporation technique, Nanoparticles, Release kinetic models

## Abstract

Nanoparticles formulated from biodegradable polymers like poly(lactic-co-glycolic acid) (PLGA) are being extensively investigated as drug delivery systems due to their two important properties such as biocompatibility and controlled drug release characteristics. The aim of this work to formulated diazepam loaded PLGA nanoparticles by using emulsion solvent evaporation technique. Polyvinyl alcohol (PVA) is used as stabilizing agent. Diazepam is a benzodiazepine derivative drug, and widely used as an anticonvulsant in the treatment of various types of epilepsy, insomnia and anxiety. This work investigates the effects of some preparation variables on the size and shape of nanoparticles prepared by emulsion solvent evaporation method. These nanoparticles were characterized by photon correlation spectroscopy (PCS), transmission electron microscopy (TEM). Zeta potential study was also performed to understand the surface charge of nanoparticles. The drug release from drug loaded nanoparticles was studied by dialysis bag method and the in vitro drug release data was also studied by various kinetic models. The results show that sonication time, polymer content, surfactant concentration, ratio of organic to aqueous phase volume, and the amount of drug have an important effect on the size of nanoparticles. Hopefully we produced spherical shape Diazepam loaded PLGA nanoparticles with a size range under 250 nm with zeta potential −23.3 mV. The in vitro drug release analysis shows sustained release of drug from nanoparticles and follow Korsmeyer-Peppas model.

## Background

Nanotechnology defines the study and production of structures and devices on a nanoscale range. Nanoparticles have been extensively studied by researchers in biomedical and biotechnological areas, especially in drug delivery systems, because their particle size they have the potential to increase drug stability, improve its duration of therapeutic effect and reduce its degradation metabolism as well as cellular uptake [1]. Preparation and characterization of nanoparticles are today’s an important task for researchers, as selection of size and shape of nanoparticles provides a capable control over many of the physical and chemical properties [2]. Different materials can be used to form these nanoparticles such as polymers, lipids, natural biopolymer etc., but biodegradable and biocompatible polymers have been widely used for the preparation of polymeric nanoparticles [3].

Poly(D,L-lactide-co-glycolide) (PLGA) polymers, have attracted significant interest for delivery systems because:

- They are biocompatible, biodegradable and less toxic [4].
- Approved by the FDA (US Food and Drug Administration) [5].
- The exclusive structure of PLGA nanoparticles, composed of a hydrophilic surface and a hydrophobic core, provides a drug carrying reservoir and also enables them to dissolve in aqueous solutions [6].
- The by-products of PLGA are the lactic acid and glycolic acid, can be excreted from the body as water and carbon-dioxide through the tricarboxylic acid cycle [7, 8].
- The most remarkable one is probably the carrier delivery system that encapsulates active ingredients and releases them under a controlled mechanism [9].

Various methods are proposed for the preparation of drug loaded PLGA nanoparticles such as an emulsion solvent evaporation method [10], nanoprecipitation method [11], double emulsion solvent evaporation method [12] etc. Many stabilizers are used to prevent the aggregation of these nanoparticles [13] and different organic solvents are used to dissolve the polymer and drug [14].

**Fig. 1:**
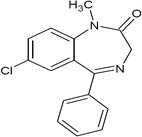
Chemical structure of Diazepam

Diazepam is a lipophilic benzodiazepine derivative drug. Benzodiazepines are considered the treatment of choice for acute management of cruel seizures. Benzodiazepines are active against a wide range of seizure types, have a rapid onset of action once delivered into the central nervous system, and are safe [15]. The IUPAC name of diazepam is 7-chloro-1,3-dihydro-1-methyl-5- phenyl-1,4-benzodiazepin-2(3*H*)-one, it is widely used as an anticonvulsant in the treatment of various types of epilepsy, insomnia, anxiety and for induction and maintenance of anesthesia [16]. Figure 1 shows the chemical structure of diazepam.

Diazepam could be administered via different routes: orally, intravenous injections, rectal solutions, rectal gels, and suppositories [17]. Generally, oral administration of Diazepam is the route of choice in the daily practice of pharmacotherapy. However, abuse of diazepam can have serious consequences, even causing death when taken in overdose [18].

On the basis of literature reviewed, it was found that several authors reported the preparation of Diazepam loaded nanoparticles with different method by using different stabilizer, but none of them previously reported the preparation and optimization of PLGA nanoparticles containing Diazepam by emulsion-solvent evaporation method by using PVA as stabilizer.

The aim of the work to report here was to design and characterize Diazepam-loaded PLGA nanoparticles in order to obtain a controlled release system. Nanoparticles were prepared by the emulsion solvent evaporation method and characterized the formulation in terms of size, morphology, drug encapsulation and drug release. This work also investigated the effect of different preparation variables such as sonication time, polymer content, organic phase volume to aqueous phase volume ratio, surfactant content and amount of drug. The in vitro drug release and the drug release data of drug loaded nanoparticles was studied by dialysis bag method and various kinetic models respectively. The significance of this work is to explain the effect of different parameters scientifically which are responsible to control the size of nanoparticles prepared by this method.

## Methods

### Materials

The biodegradable polymer studied was PLGA (RESOMER^®^ RG 504 molecular weight range is 38,000–54,000 and inherent viscosity is 0.45–0.60 dl/g) with a copolymer ratio of dl-lactide to glycolide of 50:50 gifted from Evonik Mumbai (India). The surfactant used in this process was polyvinyl alcohol (PVA) purchased from Sigma-Aldrich, Mumbai (India). Diazepam was received as gift sample from Windlas Biotech Ltd, Dehradun (India). Purified water of Milli-Q quality was used to prepare the solutions as well as the aqueous phases of the emulsions. All other reagents were of analytical grade.

### Preparation of diazepam loaded nanoparticles

The diazepam loaded nanoparticles were prepared by an emulsion solvent evaporation method [10]. Typically, known amounts of mass of PLGA polymer and diazepam were added into ethyl-acetate, which was suitably stirred to ensure that all material was properly dissolved in solvent. Then, the solution of organic phase was slowly poured into the stirred aqueous solution of PVA. This mixture was sonicated using a microtip probe sonicator energy output of 55 W in a continuous mode (Soniweld Probe Sonicator, Imeco Ultrasonics, India) for a few minutes. The formed oil in water (O/W) emulsion was gently stirred at room temperature by a magnetic stirrer (Remi, India) for 5 hours to evaporate the organic solvent. The nanoparticles were recovered by centrifugation (22,000 rpm, 25 min; WX ultra 100 ultracentrifuge Thermofisher Scientific USA) and washed with distilled water 2–3 times to remove the surfactant. The purified nanoparticles were freeze-dried (YSI-250, Yorco Freeze Dryer (Lyophilizer), Yorco Sales Pvt. Ltd., India) to obtain the fine powder of nanoparticles, which was placed and kept in vacuum desiccators.

### Nanoparticles characterization

The size (Z-average mean) and zeta potential of the nanoparticles were analyzed by photon correlation spectroscopy (PCS) or dynamic light scattering (DLS), respectively, in triplicate using a Zetasizer (Model- ZEN 3600, Malvern Instruments, U.K.). The dried powder samples were suspended in distilled water and slightly sonicated before analysis. The obtained homogeneous suspension was measured for the volume mean diameter and size distribution. Each measurement was done in triplicate. The shape, surface morphology and size analysis of the nanoparticles were analyzed by transmission electron microscopy (TECNAI 200 kV TEM (Fei, Electron Optics) Japan). A droplet of the nanoparticles was placed on a carbon-coated copper grid, forming a thin liquid film. The negative staining of samples was obtained with a 2 % (w/V) solution of phosphotungstate acid.

### Entrapment efficiency

Nanoparticles were separated from dispersion by centrifugation at 22,000 rpm for 25 min. The supernatant obtained after centrifugation was suitably diluted and analyzed for free diazepam by UV–Visible spectrophotometer (Model No.-2201, UV–visible double beam spectrophotometer, Shimadzu, India) at 325 nm. The percentage entrapment efficiency was calculated as:

1 $$\% {\text{ Entrapment efficiency}} = \frac{{\left[ {Drug} \right]total - \left[ {Drug} \right]supernant }}{{\left[ {Drug} \right]total}}\varvec{ } \times 100$$

### In-vitro drug release

The in-vitro drug release study of diazepam loaded PLGA nanoparticles formulations were studied by dialysis bag diffusion method [19]. Drug loaded nanoparticles (5 ml) were dispersed into dialysis bag and the dialysis bag was then kept in a beaker containing 100 ml of pH 7.4 phosphate buffer. The beaker was placed over a magnetic stirrer and the temperature of the assembly was maintained at 37 ± 1 °C throughout the experiment. During the experiment rpm was maintained at 100 rpm. Samples (2 ml) were withdrawn at a definite time intervals and replaced with equal amounts of fresh pH 7.4 phosphate buffer. After suitable dilutions the samples were analyzed using UV–Visible spectrophotometer at 325 nm.

To analyze the in vitro drug release data various kinetic models were used to describe the release kinetics. The zero order rate Eq. (2) explains the systems where the rate of drug release does not depend on its concentration [20]. The first order Eq. (3) explains the release from the system where rate of drug release is concentration dependent [21]. Higuchi [22] described the release of drugs from insoluble matrix as a square root of time dependent process based on Fickian diffusion Eq. (4). Korsmeyer et al. [23] derived a simple mathematical relationship which described the drug release from a polymeric system Eq. (5).

2 $$C \, = \, k_{o} t$$

where, C is the concentration of drug at time t, t is the time and k_0_ is zero-order rate constant expressed in units of concentration/time.

3 $$Log \, C_{0} {-} \, Log \, C \, = \, k_{1} t / 2.303$$

where, C_0_ is the initial concentration of drug and k_1_ is the first order rate constant.

4 $$C \, = \, K_{H} \sqrt t$$

where, K_H_ is the constant reflecting the design variables of the system.

5 $$M_{t} / \, M_{\infty } = \, K_{KP} t^{n}$$

where M_t_/M_∞_ is the fraction of drug released at time t, K_KP_ is the rate constant and n is the release exponent.

## Results and discussion

### Effect of different preparation variables on formulation characteristics

By using the emulsion solvent evaporation technique, several process parameters were tested to achieve best preparation conditions, including time of sonication, amount of polymer in the formulation, surfactant content in the formulation, organic to aqueous phase volume ratio and diazepam content. Only one factor was replaced in each series of experiments.

#### Effect of sonication time

**Fig. 2:**
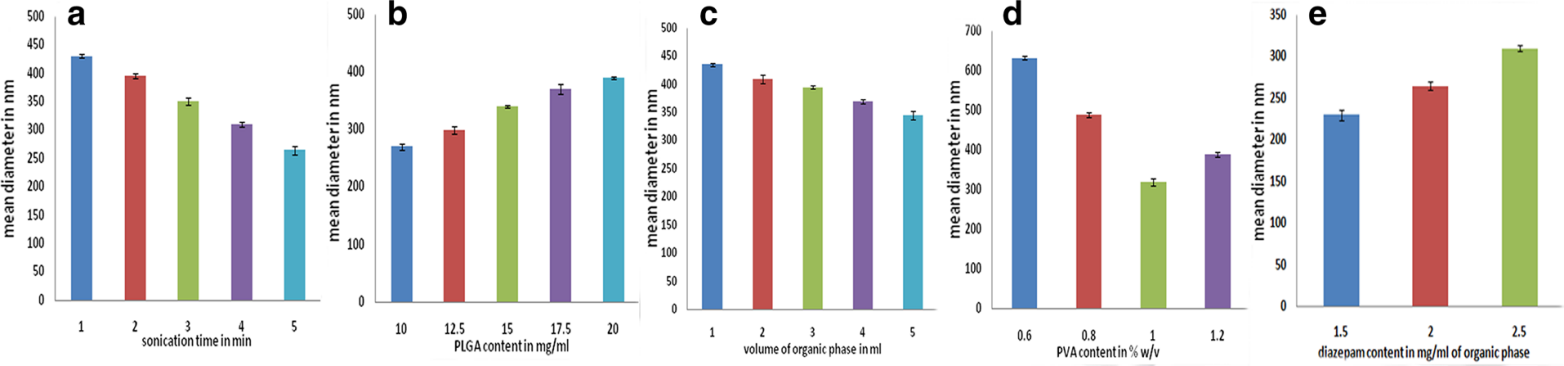
Effect of **a** sonication time on the size of nanoparticles **b** PLGA content in organic phase in mg/ml on the size of nanoparticles **c** volume of organic phase in ml on the size of nanoparticles **d** volume of PVA content in  %w/V of aqueous phase and **e** diazepam content in mg/ml of organic phase

**Fig. 3:**
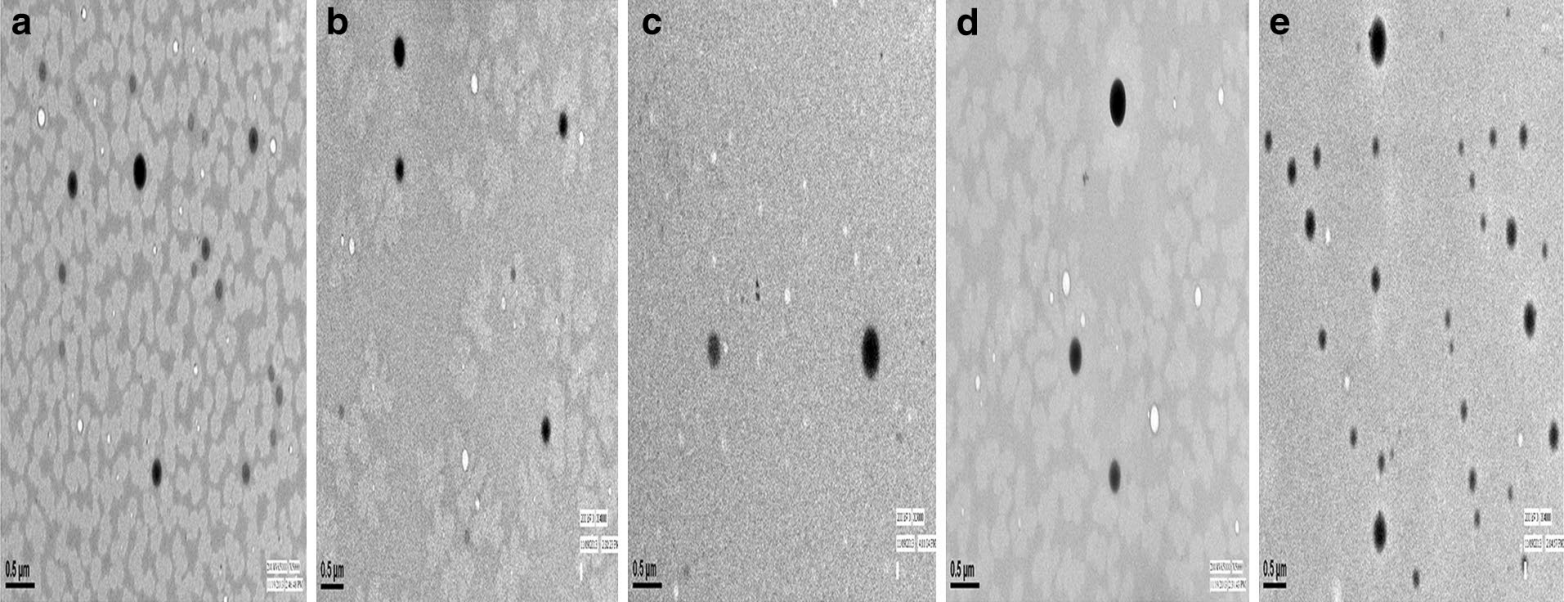
TEM image for nanoparticles prepared by **a** 5 min sonication **b** 10 mg/ml PLGA of organic phase **c** 5 ml of organic solvent **d** 1 % (w/V) PVA of aqueous phase and **e** 1.5 mg/ml diazepam of organic phase (or optimization o different variables)

In emulsion solvent evaporation technique the fundamental step is the addition of energy to obtain the emulsion and it is provided by sonication. To explain the influence of sonication time on nanoparticles shape and size, it was varied from 1 to 5 min. The preparation procedure yielded spherical particles in all cases according to TEM results. It can be concluded that on increasing the sonication time (from 1 to 5 min) the applied energy increases, so this leads to a decrease in the size of nanoparticles (from 430 to 265 nm), these results are summarized by in Fig. 2 by graph (a) and TEM image for nanoparticles prepared by 5 min sonication in shown in Fig. 3 by image (a). The emulsification can be considered one of the most significant steps of this technique, because the formation of large particles is the outcome of an insufficient dispersion of phases. Our results are in accordance with those observed by other authors [24, 25].

#### Effect of PLGA content

To verify the influence of the effect of polymer content, it was varied between 10 to 20 mg/ml of organic phase, and the effect of the initial amount of polymer on the particles morphology and size were studied. The results are concluded by graph (b) in Fig. 2.

According to TEM results nanoparticles prepared with different amount of polymer are spherical in shape, but on increasing the polymer content lead to a regular increase in nanoparticles diameter. When the amount of PLGA was doubled from 10 to 20 mg/ml of organic phase, the particle diameter increased from 270 to about 390 nm. In Fig. 3 image (b) exhibits the TEM image of nanoparticles prepared by using the PLGA as 10 mg/ml of organic phase. According to this result, it can be conclude that, for this technique the polymer content in the organic phase is a significant factor, because the size of nanoparticles increased as polymer concentration was increased. The increase in the particle size with an increase in polymer amount was reported by other authors [26, 27]. This was probably caused by the increasing viscosity of dispersed phase (organic phase), resulting a low dispersability of the PLGA solution into the aqueous phase. Increase in polymer concentration leads to an increase in the viscous forces resisting the droplet break down by sonication. These forces oppose the shear stresses in the organic phase and the final size of particles depends on the net shear stress, which is available for droplet breakdown [24].

#### Effect of organic phase volume to aqueous phase volume ratio

The ratio of organic phase and aqueous phase of an emulsion is an important factor in this technique. The organic volume was varied among 1–5 mL by keeping constant the aqueous phase volume, and its effect on nanoparticles size was observed and summarized in graph (c) in the Fig. 2 and TEM result by image (c) in Fig. 3. From the results it was observed that an increase in the organic/aqueous ratio leads to decrease of the size of nanoparticles from 430 to 345 nm. This occurs due to the coalescence of droplets can be prevented by a large amount of organic solvent available for diffusion in the emulsion.

#### Effect of PVA content

To study the effect of PVA content on the nanoparticles, the aqueous phase with different PVA content (0.6 to 1.2 % w/V) was prepared. It can be noticed that as the PVA content is increased, the diameter of nanoparticles, first decreases and then gradually increases, the results are shown in graph (d) in Fig. 2 and TEM image (d) in Fig. 3. The presence of PVA molecules stabilizes the emulsion nanodroplets and prevents them from aggregation with one another. For a better stabilization, the surfactant molecules must cover the organic/aqueous interfacial area of all the droplets. Hence a lowest amount of PVA molecules is required to achieve small size of nanoparticles. As the concentration of PVA is increased, the size of particles produced by this method decreases and then increases due to the increased viscosity of the aqueous phase; the viscosity increase reduces the net shear stress available for droplet breakdown (which is already discussed in “Effect of PLGA content” section). So the size decreases due to enhanced interfacial stabilization whiles it increases due to the increased aqueous phase viscosity. The amount of surfactant plays an important role in this technique [28, 29].

#### Effect of diazepam content

In this section the effect of diazepam into PLGA nanoparticles was examined. Maintaining constant all other formulation variables, the amount of diazepam used was varied 1.5 to 2.5 mg/ml of organic solvent. It can be concluded that the increase in the initial amount of drug increases the size of nanoparticles from 230 to 310 nm; results are summarized by graph (e) in Fig. 2. This can be explained by the fact that a greater amount of drug results in a more viscous organic phase (dispersed phase), making complex the mutual dispersion of the phases and forming bigger nanoparticles. TEM experiments showed that the particles remained with a spherical shape in all cases, in Fig. 3 image (e) shows the TEM picture of nanoparticles prepared by drug in amount 1.5 mg/ml of organic solvent.

**Fig. 4:**
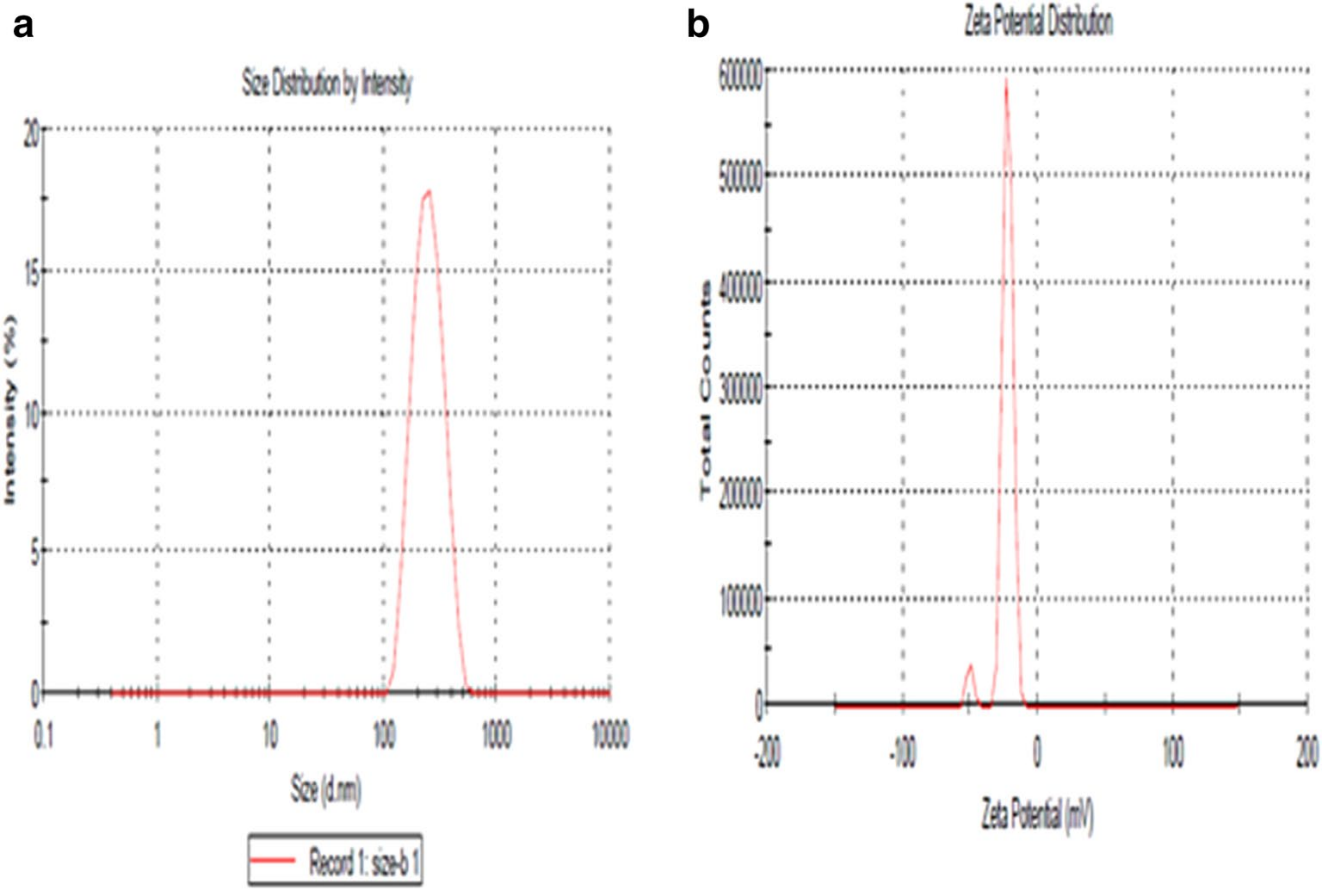
**a** DLS image and **b** Zeta potential graph for nanoparticles prepared by optimization of different variables

On the basis of the above discussion optimized diazepam loaded-PLGA nanoparticles were successfully prepared in the size range 230 nm. For this purpose, optimized variables are as: PLGA 10 mg/ml of organic phase, PVA 1 % w/V of aqueous phase, 5 ml of ethyl-acetate was used as organic solvent, diazepam used as 1.5 mg/ml of organic phase and sonication time was 5 min. By this optimized formulation we had got spherical nanoparticles with a size range in 230 nm and zeta potential as −23.3 mV and drug entrapment efficiency as 66 %. TEM image of these nanoparticles is shown in Fig. 3(e), zeta size image and zeta potential of these nanoparticles in Fig. 4.

### In-vitro drug release

**Fig. 5:**
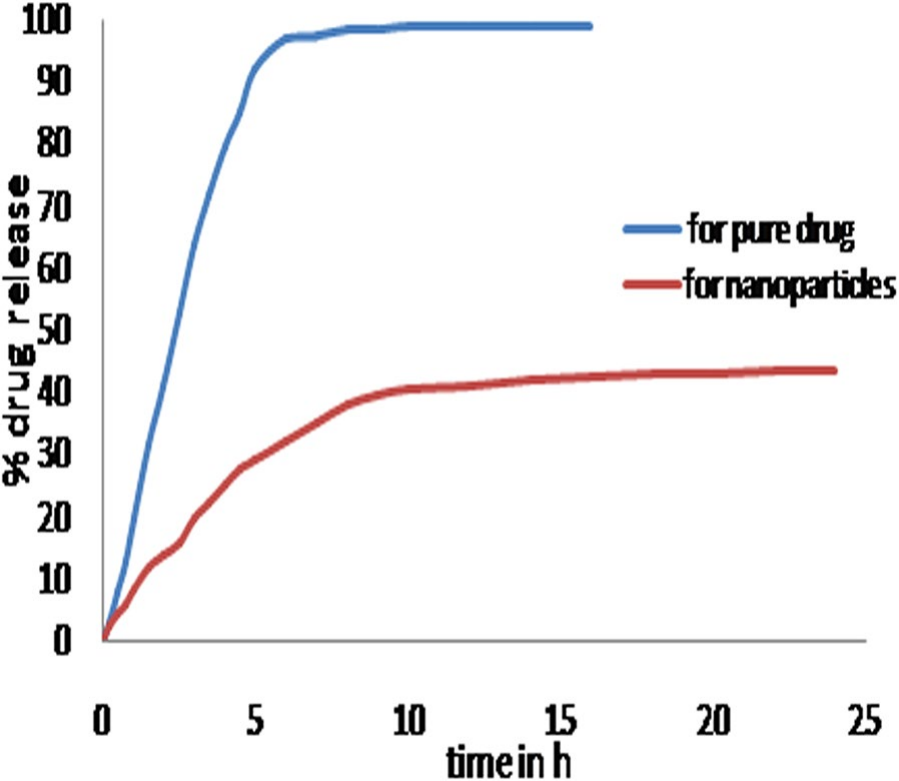
In-vitro drug release for nanoparticles prepared by optimization of different variables

**Fig. 6:**
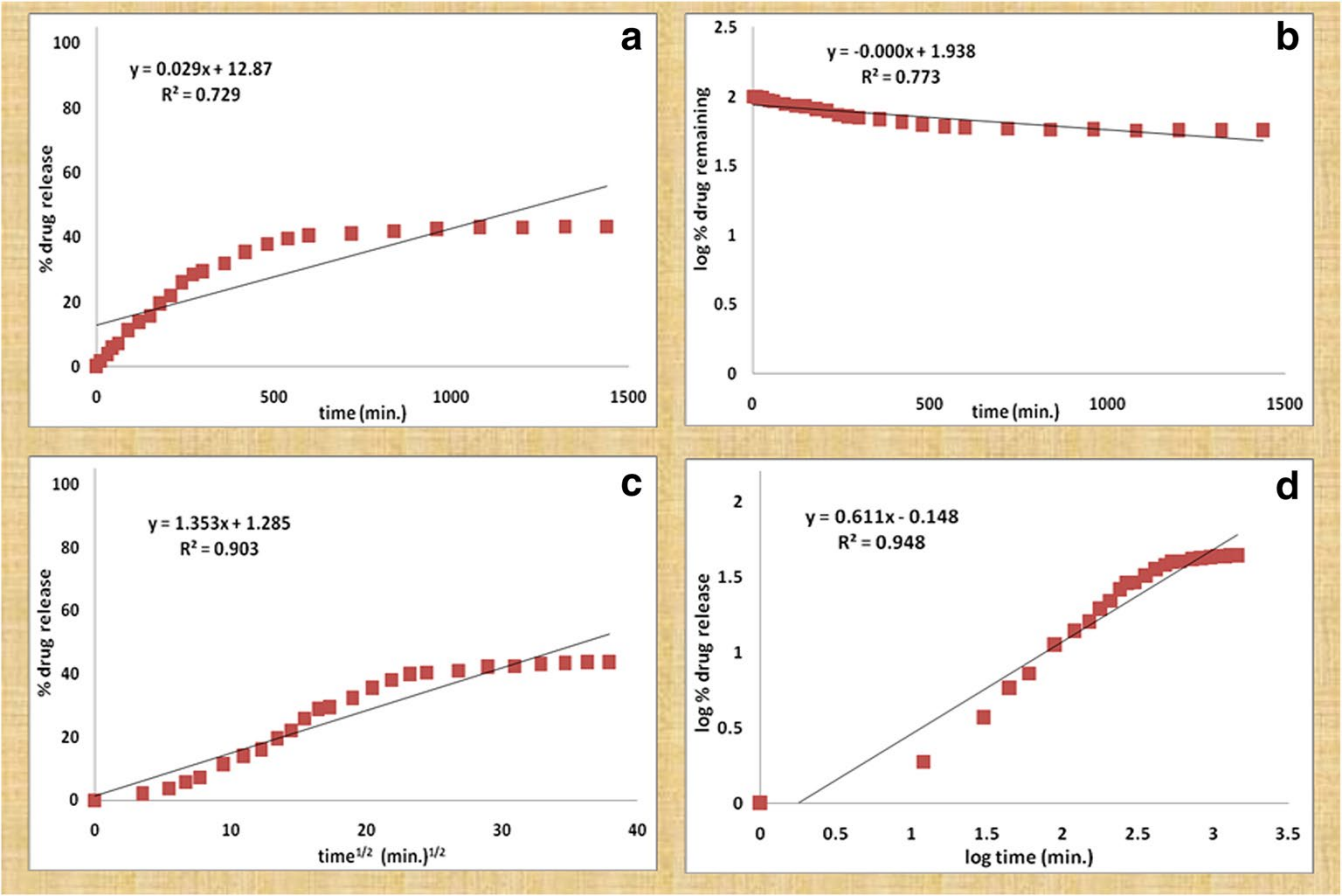
Drug release kinetics plots: **a** Zero order plot **b** First order plot **c** Higuchi plot and **d** Korsmeyer Peppas plot

In-vitro drug diffusion studies were carried out using dialysis bag method. The data of percentage drug release formulation were shown in Fig. 5. For kinetic study following plots were made: cumulative  % drug release vs. time (zero order kinetic model); log cumulative % drug remaining vs time (first order kinetic model); cumulative  % drug release vs square root of time (Higuchi model); log cumulative  % drug release vs log time (Korsmeyer–Peppas model). All Plots are shown in Fig. 6 and results are summarized in Table 1. In the above table R^2^ is correlation value, k is rate constant and n is release exponent. On the basis of best fit with the highest correlation (R^2^) value it is concluded that in the optimized formulation of nanoparticles follow the Korsmeyer-Peppas model with release exponent value n = 0.61. The magnitude of the release exponent n indicates the release mechanism is non Fickian diffusion.

**Table 1:** Interpretation of R-square values and rate constants of release kinetics of nanoparticles

Model	R^2^	K	n
Zero order	0.729	1.1308 × 10^−1^	–
First order	0.773	3.9216 × 10^−1^	–
Higuchi	0.903	1.2588	–
Korsmeyer-Peppas	0.948	1.5848 × 10^−4^	0.61

## Conclusions

From the above investigation, we can conclude that the preparation of drug loaded nanoparticles by emulsion solvent evaporation method is governed by different preparation variables. By the systematic study of these variables, we got the valuable results. In this technique the most important factor for reducing the size of nanoparticles is increase the shear stress during emulsification, which is done by increasing the applied energy, decreasing the polymer content in organic solvent, using the sufficient amount of surfactant, increasing the organic phase volume to aqueous phase volume ratio. On the basis of optimization of these variables we have successfully synthesized the spherical nanoparticles of diazepam which are reproducible.

## Abbreviations

PLGA: poly(lactic-co-glycolic acid)
PVA: polyvinyl alcohol
TEM: transmission electron microscopy
